# Plant-Based Films for Food Packaging as a Plastic Waste Management Alternative: Potato and Cassava Starch Case

**DOI:** 10.3390/polym16172390

**Published:** 2024-08-23

**Authors:** Luna Valentina Angulo Arias, Viviane de Souza Silva, Jorge Miguel Magalhães Vieira, Farayde Matta Fakhouri, Rafael Augustus de Oliveira

**Affiliations:** 1 School of Agricultural Engineering, University of Campinas, Campinas 13083-875, SP, Brazil; lunaangulo7@gmail.com (L.V.A.A.); vivianesouzasilva81@gmail.com (V.d.S.S.); 2 School of Food Engineering, University of Campinas, Campinas 13083-862, SP, Brazil; jorgevieirabcd@gmail.com; 3 Poly 2 Group, Department of Materials Science and Engineering, Polytechnic University of Catalonia, 08019 Terrassa, Spain

**Keywords:** biopolymers, edible films, plastic pollution, sustainability

## Abstract

The escalating environmental impact of plastic packaging waste necessitates sustainable alternatives in food packaging. This study explores starch-based films derived from cassava and potato as viable substitutes, aiming to mitigate plastic pollution and enhance environmental sustainability. Utilizing a casting method, formulations optimized by CCRD were characterized for their physical, physicochemical, and morphological properties. Comprehensive analysis revealed both cassava and potato starch films to exhibit robust structural integrity, high tensile strength (up to 32.6 MPa for cassava starch films), and semi-crystalline morphology. These films demonstrated low water vapor permeability and moderate solubility, akin to conventional low-density polyethylene used in packaging. Differential scanning calorimetry indicated glass transition temperatures between 116.36 °C and 119.35 °C, affirming thermal stability suitable for packaging applications. Scanning electron microscopy confirmed homogeneous film surfaces, with cassava starch films (C4-15) exhibiting superior transparency and uniformity. X-ray diffraction corroborated the films’ semi-crystalline nature, unaffected by sorbitol content variations. Despite their mechanical and thermal suitability, further enhancements in thermal degradation resistance are essential for broader thermoprocessing applicability. These findings underscore the potential of starch-based films to be used as lids or other part of a food package, decreasing the plastic dependency in food packaging, contributing decisively to waste reduction and environmental preservation.

## 1. Introduction

Currently, the population is being more conscious about responsible consumption, concerning on how food is produced and how it affects health, household budget, and the environment. Urbanization and industrialization increase food waste generation and, to avoid and reduce waste pollution or any negative impact, it is necessary to develop innovative techniques for waste management in order to reduce their volume or transform them into some valuable or commercially viable products [1,2]. The plastic production industry, its mismanagement, and the indiscriminate use of plastics have resulted in a global pollution problem; this material is degrading into micro(nano)plastics in the environment. Micro(nano)plastic pollution is widespread and has caused widespread ecological impacts around the world, threatening sustainability. At the same time, an alarm has been generated in the international community that highlights the need for a more holistic approach to reduce plastic and micro(nano)plastic pollution and, so far, few studies have reported pollution-preventive actions, mitigation, or elimination techniques of micro(nano)plastics [3]. The increase in the waste of plastic food packaging is a factor with a high negative impact in all industrialized countries, and the implementation of sustainable viable solutions is urgent [4,5].

Reuse, reducing, and recycling of waste are the most powerful and effective tools for effective waste management. Whence, it is imperative to mitigate plastic pollution, find alternative uses for waste, and develop new products that can substitute plastic packaging in industry [5,6,7]. Those considerations could be underpinned by the circular economy principles, keeping products and materials in use, and regenerating natural systems, looking for a responsible production of food that improves rather than degrades the environment, so that the population can access healthy and nutritious food [8].

Plastic packaging materials used for food have a negative environmental impact, affecting mainly the marine trophic chain. Recently, the number of studies related to profitable manufacturing methods that allow the development of environmentally friendly products, mainly those called bioplastics, has increased. Thus, it is expected that more methods will be applied at an industrial level, reducing the impact of discarding products such as food packaging [9,10,11].

Therefore, developing new products from by-products with functional properties and added value ensures the total productive chain exploitation being a responsible production practice and good management of the environmental pollution problem.

Given the increasing health concerns, environmental impacts, and the importance of food quality, biopolymeric materials such as edible and biodegradable starches offer a promising alternative to plastic for food packaging. These materials combine desirable properties, including a higher degradation rate, making them an attractive and sustainable option [12,13,14].

The application of edible films has increased rapidly resulting in maintaining the quality of different food products, innovating in solving quality problems related to the food supply chain during transportation and storage, including shelf life in supermarkets and warehouses before arriving to the final consumer [15,16].

Biopolymers could be used to develop innovative materials such as edible, intelligent, and antimicrobial films suitable for food packaging development in replacement of plastic materials. Edible films must meet specific parameters for this type of application, and starch is one of the natural polymers capable of forming edible films [17]. An example of innovative intelligent biopolymer based material is an indicator film based on whey protein isolate nanofibers, anthocyanin, and glycerol that was used to detect salmon freshness [18] and, as antimicrobial film example, in a recent study, red propolis was added to biodegradable protein film which showed antimicrobial activity against *Staphylococcus aureus* and *Pseudomonas aeruginosa* [19]; in another case, the incorporation of copaiba oil in starched-based films successfully inhibited Gram-positive bacteria [20]. In 2021, starch represented 16.4% of global bioplastic production, highlighting its importance as a sustainable and renewable raw material, despite the future work required to make biologically based and biodegradable materials profitable [11].

The packaging technique, combined with materials with appropriate gas and water barrier properties, aims to protect food from microbial and insect degradation [5]. Although, starch-based materials have not been used widely for food packaging because of their hydrophilic behavior, low gas barrier, and water sensitivity [21]. Consequently, testing different starch types and different film formulations is an alternative to generate the knowledge needed to improve the development of starch-based materials that are edible, biodegradable, and suitable for food packaging. Cassava starch is a widely available biopolymer and cost-effective material. In the last five years, it has been the subject of many studies, with Brazil and China leading the starch-based films research. Those previous studies showed that cassava starch is an edible ingredient, and the films developed were biodegradable, although the authors have suggested that modifications are required to obtain a more structurally integrated matrix [22]. Yet, compared to other starch types, the potato-processed starch is considered pure with properties attributed to its granular and molecular structures, as very large and smooth granules, a high content of covalently linked phosphate, long amylopectin chains, and high-molecular-weight amylose make it unique. These characteristics combined make potato starch a tremendous source of functional biopolymer for food and materials [23].

Given this context, the present work aims to optimize the formulation of cassava and potato starch-based films using a central composite rotatable design and characterize the optimal formulated films according to their physical, physicochemical, and morphological properties using scanning electron microscopy (SEM), X-ray diffraction (XRD) and differential scanning calorimetry (DSC) analyses.

## 2. Materials and Methods

### 2.1. Material

Potato starch (PoS) (allotment G18BRPP241, Yoki, São Bernardo do Campo-SP, Brazil) and cassava starch (CaS) (allotment EA163, Casa do Naturalista, Amparo-SP, Brazil) from a local market were used for the edible films’ formulations.

### 2.2. Methods

#### 2.2.1. Starch Films Formulations for Food Packaging

An experimental central composite rotatable design (CCRD) with 4 axial and 3 central points was used to develop predictive mathematical models to optimize starch film formulations. A total of 11 experiments (Table 1) to predict water vapor permeability, thickness, water content, solubility, and water activity as a function of two independent variables, the concentration of starch and concentration of plasticizer, related to the starch concentration.

#### 2.2.2. Preparation of the Edible Films

The films were produced using the casting method. Filmogenic solutions were obtained according to the 11 experimental run values in Table 1. The plasticizer used was liquid sorbitol at 70%, and the real concentration of “y” considered was related to the value of real “x”. Starch was hydrated with distilled water and disposed into a thermostatic bath (Dubnoff, Solab SL-157, Piracicaba-SP, Brazil) at 80 °C for 270 s with constant manual agitation. Then, sorbitol was added and homogenized. To 150 mm × 15 mm petri dishes, 27 mL of filmogenic solution was added and arranged on a level table in a room with an air conditioning system at 25 °C for drying [12]. Dried times for PoS and CaS films were 48 h and 36 h, respectively.

#### 2.2.3. Water Content and Solubility Analyses

The samples were conditioned to relative humidity (RH) control (55% ± 3 of RH) for two days prior to analysis. Water content was determined by drying at 105 °C in a stove without air circulation until constant weight (320/3, Fanem, Guarulhos-SP, Brazil) and calculated by weight difference [24]. Then, the dried samples were immersed in distilled water at 25 °C for 24 h with 115 rpm of agitation, then dried at 105 °C without air circulation until constant weight, and the solubility was calculated by the dry matter difference [25].

#### 2.2.4. Water Vapor Permeability (WVP)

WVP was measured according to ASTM standard E96/96M-13, with adjustments using acrylic cells sealed with O-rings and acrylic lids fixed with screws with an opening in the center. The mass gain of calcium chloride was measured to determine the water vapor transfer, which was measured every 60 min until 12 measurements were completed [26]. Thickness measurements were expressed as the mean of 15 measurements taken with a digital micrometer (MDC Lite 293-821-30, Mitutoyo Sul Americana, Jundiaí-SP, Brazil) [27]. Thickness measurements of the WVP were also used as a value for the analysis of the films thickness.

#### 2.2.5. Water Activity (a_w_)

Water activity was measured using a water activity meter (Aqualab 4TE, Aqualab, Pullman, WA, USA) by a direct reading of 3.6 cm disks of starch films with samples conditioned at 55% ± 3 RH for two days before analysis.

#### 2.2.6. Mechanical Properties

The tensile strength and elongation at break were measured by a texture analyzer (TA.TX Plus Texture Analyser, Stable Micro Systems, Surrey, UK) with A/TG tensile grips and using “Texture Exponent 32” software (Stable Micro Systems, Surrey, UK), according to Standard Method D882–12, with adjustments [28]. Film samples were cut into strips (2.5 cm wide, 10 cm long) and tested with an initial grip separation of 8 cm and a crosshead speed of 1.0 mm/s. Tensile strength (force/initial cross-sectional area) and elongation at break were computed directly from the curves of strength vs elongation curves using “Texture Exponent 32” software. Young’s modulus was calculated from the initial linear portion of the strength vs elongation curve [29].

#### 2.2.7. Scanning Electron Microscopy (SEM), X-ray Diffraction (XRD), and Differential Scanning Calorimetry (DSC)

Two days before analyses, film samples (2 cm × 2 cm) were conditioned at 55% ± 3 RH.

SEM analysis was conducted by breaking samples with liquid nitrogen, mounting fragments on aluminum stubs with double-sided tape, and coating with gold. The samples were viewed under a scanning electron microscope (LEO 440 i, Cambridge, UK) at 15 kV. XRD analysis was performed using a diffractometer (X´Pert-MPD, Philips Panalytical X-ray, Almelo, Netherlands) with a Cu target, scanning from 5° to 70° (2θ) at 0.0166 °/s. DSC measurements were carried out using a calorimeter (DSC1, Mettler Toledo, Schwerzenbach, Switzerland) with a liquid nitrogen cooling module. The tests were heated from 25 °C to 160 °C at a rate of 10 °C/min. Atmospheric air was the reference material.

#### 2.2.8. Statistical Analysis

All experiments were performed randomly, and data were treated by software STATISTICA^®^, version 9.0, to calculate the analysis of variance (ANOVA). Tukey tests were used to determine the difference between the means with a confidence interval of 95%.

## 3. Results and Discussion

### 3.1. Experimental Results of Physicochemical Characterization

The CCRD experimental design allowed us to analyze some parameters of different plant-based films from potato and cassava starch (PoS and CaS) and find out which formulation better meets the requirements of edible films. Based on the experimental design, the results for PoS films (Table 2), and the statistical analysis (Table 3), mathematical models were calculated using the encoded values of x and y for each parameter and expressed by Equations (1)–(4).

(1) $$Water\;content=19.9-5.8x-3.08xy\;R^{2}=0.91$$

(2) $$Aw=0.51-0.042x-0.022x^{2}\;R^{2}=0.88$$

(3) $$WVP=5.83-1.21x^{2}-0.41y+0.56xy\;R^{2}=0.70$$

(4) $$Thickness=0.044+0.012x-0.003y-0.004xy\;R^{2}=0.94$$

Solubility showed one significant factor, and R^2^ = 0.55; so, it was considered that the model did not describe the experimental solubility results for PoS films.

**Figure 1:**
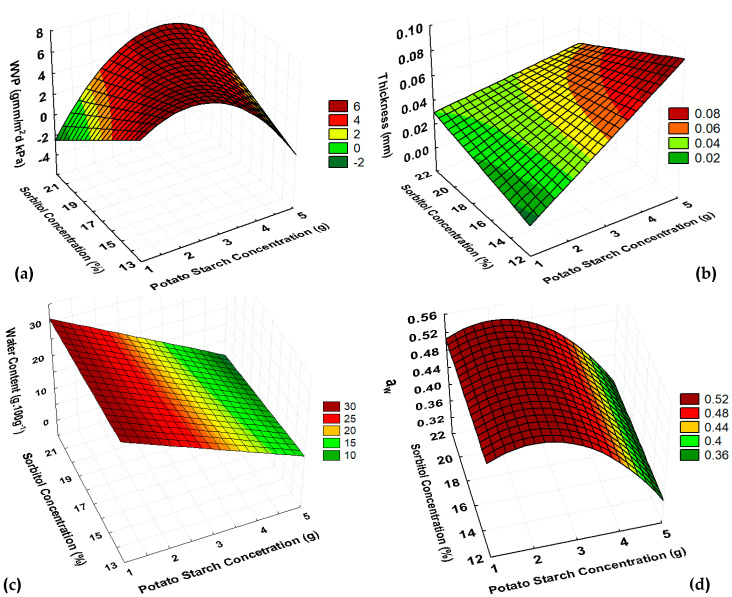
Response surfaces for (**a**) WVP, (**b**) thickness, (**c**) water content, and (**d**) a_w_ of PoS films.

From Figure 1a, it was noticed that the increasing of PoS concentration tended to increase the films WVP, and increasing the sorbitol percentage tended to decrease the WVP.

The quadratic relation between starch concentration and sorbitol percentage confirmed that as well the individual influence of each factor, their interaction also affected the WVP behavior. So, the lowest WVP values occurred when the highest starch concentration and the central point of sorbitol interacted.

Low WVP are desirable for packaging material. Thus, previous studies showed that a high starch concentration related to the same concentration of sorbitol resulted in films with lower WVP [30]. Some studies have reported WVP values for both native and modified potato starch films ranged from 5 to 10 g·mm/m^2^·day·kPa. Nevertheless, lower values can also be achieved, such as the 4.22 g·mm/m^2^·day·kPa that was reported for gelatine with native potato starch films [30,31]. According to Table 2, WVP values ranged from 2.68 to 6.80 g·mm/m^2^·day·kPa, reaching values within and below the previous studies.

For the thickness of PoS film, the results corroborated what was previously reported [32]. The positive linear relation between thickness and starch content evidenced that thickness is directly related to starch concentration (Figure 1b). When compared to other studies, the thickness results were lower (0.024 to 0.068 mm) since the previous reports showed values ranging from 0.06 to 0.168 mm [31,32].

PoS showed a negative linear relation between water content and starch concentration. The addition of sorbitol did not influence its water content (Figure 1c). So, at higher starch concentrations, films presented lower water content, which is a desirable property of edible films to inhibit the development of degradative microorganisms that affect food quality. Another important parameter for an edible film to be microbiological stable is the a_w_ values, which showed a positive quadratic relation with starch concentration for PoS without a sorbitol percentage influence (Figure 1d). Thus, starch concentration in PoS film will affect the available water for the physiological process involved in food packaging.

PoS films’ solubility varied from 3.13 to 24.04 g·100 g^−1^. Those values were lower than 27.53% of potato starch with gelatine films and closer to the values reported for native potato starch films, which ranged from 14.26 to 19.87% [30,31]. Although solubility is not statistically representative data, it is important to analyze its behavior as it influences the disposal of the material and its biodegradation due to environmental conditions. High solubility means that the material is easily degraded in environments with high relative humidity and that it is not suitable for food packaging of products stored in conditions in which the differences between the relative humidity of the product and the environment are high, as the physicochemical properties of the food are affected.

Thus, by the surface responses near the 4 g·100 g^−1^ of water of starch and 15% sorbitol, it was possible to observe optimal conditions of high WVP, good thickness, low water content, and low a_w_ (Table 2; Figure 1). However, it is not recommended to work with higher concentrations of potato starch for the casting method because operational difficulties such as uneven surfaces and longer drying times occur. Therefore, for the next step of the study, PoS films with concentration of starch about 4 g·100 g^−1^ of water and 15 and 20% sorbitol were evaluated according to their mechanical properties.

For CaS films, the experimental results showed that for thickness, the starch concentration was significant, but no more parameters were significant, and the value of 0.44 for R^2^ led to disregard of the mathematical model obtained. For the a_w_ of CaS films, starch concentration and sorbitol percentage effects had no statistical significance, and even when the water content showed significance, the R^2^ value was under 0.5, and a lack of fit was present. Thus, by the responses obtained with the experimental design, WVP and solubility parameters were considered to evaluate the optimal formulation for CaS. The mathematical models are presented in Equations (5) and (6) (in coded values), and both models presented R^2^ values equal to 0.8. The mathematical model for WVP had a lack of fit, but the effects were statistically significant, and the mean relative deviation was 0.145, considered low under those conditions (Table 4).

(5) $$WVP=2.42-0.87x+0.84x^{2}-0.86y+0.66+1.02xy$$

(6) $$Solubility=3.96+2.63x^{2}+2.03y+5.77y^{2}$$

**Figure 2:**
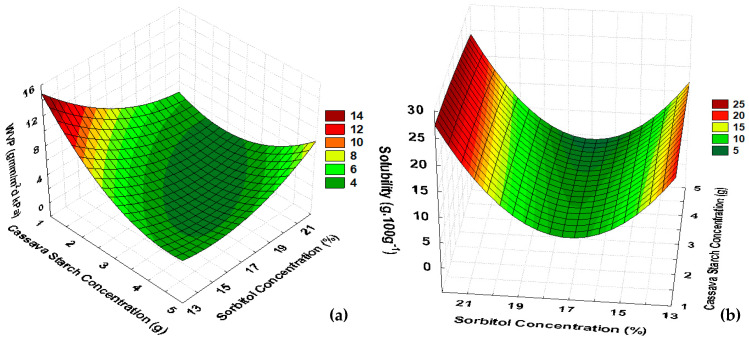
Response surfaces for (**a**) WVP and (**b**) solubility of CaS films.

According to Table 2 for CaS, the values of WVP ranged from 2.27 to 8.02 g.mm/m^2^ day.kPa, related to a negative quadratic association with the starch concentration, as shown in Figure 2a.

For films from cassava starch added with gelatine and sorbitol, two types of starch were evaluated; for native starch, WVP ranged from 3.91 to 7.45 g·mm/m^2^·day·kPa, and for modified starch, WVP ranged from 4.57 to 6.64 g·mm/m^2^·day·kPa [12]. So, the WVP of CaS matches the previous reports.

The variation in the thickness of CaS coincided with the results obtained by [12], with thicknesses from 0.034 to 0.050 mm for films with 3–5 g starch concentration.

CaS water content ranged from 4.10 to 15.47 g·100 g^−1^, lower values than PoS films, which allow us to infer that the different structures of each starch confer different water interaction. Then, potato starch had higher water binding capacity after gelatinization; therefore, drying time for potato starch films was longer.

CaS solubility showed a negative quadratic relation with sorbitol percentage, ranging from 3.06 to 18.39 g·100 g^−1^, but starch concentration did not influence the solubility. So, according to Figure 2b, from 15 to 20% of sorbitol would result in films with lower solubility.

Comparing PoS and CaS, CaS presented lower solubility values than PoS films, which is another indication of the low hydrophilic characteristic of cassava starch under the operating parameters used in this work, compared to potato starch. Then, because of the low solubility of CaS, it may be suitable for food packaging of products stored in conditions in which the differences between the relative humidity of the product and the environment are high, when compared to PoS. Higher solubility values were obtained with films made from cassava starch, with values ranging from 17.74 to 39.41%, due to the addition of gelatine, glycerol, oregano essential oil, and pumpkin residues in the formulation [12,33]. A way to improve the CaS solubility could be the addition of fruit pulp as reported by [34] that reached solubility values between 16.8 and 52.9%, whereby at higher concentrations of pulp, there was lower solubility.

The surface evaluation for WVP, considering the statistical analyses and mathematical models, highlights optimal conditions for films of cassava starch in the central region considering concentrations of cassava starch from 3 to 4 g·100 g^−1^ of water with a sorbitol percentage higher than 13% (Table 2; Figure 2a). However, a high percentage of sorbitol (above 20%) influenced the solubility, increasing the percentage of solubility and producing an unsuitable material for food packaging (Figure 2b). So, to define the optimal formulation for CaS films for food packaging, experimental runs 3, 4, and 9 were evaluated. Those experiments corresponded to the formulations with starch concentration of 4 g·100 g^−1^ of water with 15 and 20% of sorbitol corresponding to the central point in the experimental design.

### 3.2. Mechanical Properties’ Results

For food packaging materials, it is necessary to evaluate their mechanical properties, as only sufficiently strong films can withstand high external forces and perfectly protect their content [35].

**Figure 3:**
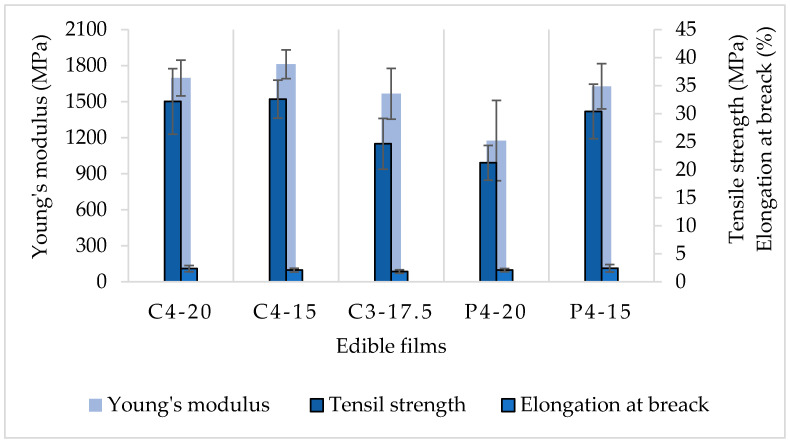
Mechanical properties of CaS and PoS films. C = CaS; P = PoS. The first number next to the capital letter indicates the starch concentration (g) and the second number indicates the sorbitol percentage (%). The columns represent the means values of the mechanical properties and the bars represent the standard deviation.

For the mechanical properties analyses, just the formulations with better physicochemical conditions were subjected to tensile strength and elongation at break (E%) evaluations (Figure 3).

The starch molecular chain flexibility marks the elongation at break condition and can be reduced by increasing the starch concentration, and it can be observed in Figure 3 where, statistically, there were no significant differences in the E% values of the evaluated films. The starch concentration of the films was 3 and 4 g·100 g^−1^ of water, with values ranging from 1.9 to 2.4%, indicating the rigid condition of the CaS and PoS films.

E% values for cassava starch films with gelatine and sorbitol, that ranged from 3.81 to 11.52%, were previously reported, demonstrating that gelatine addition could improve rigid cassava starch films conditions [12]. On the other hand, for potato starch with gelatine films, 2.86 ± 1.20% of E% was reported, similarly to CaS and PoS results [30]. So, the addition of gelatine did not improve potato starch films’ flexibility but the addition of glycerol significantly increased the elongation at break with values from 58.33 to 85.20% and, at the same time, decreased tensile strength (from 3.53 to 5.25 MPa) and Young’s modulus (YM) (from 3.95 to 9.23 MPa) [31]. Glycerol was also used for different types of potato starch films and its reported tensile strength (TS) varied from 2.0 to 2.4 MPa. E% values ranged from 25 to 32% [11], similar to those reported by [35]. Glycerol addition could improve some mechanical properties, but for oxidized potato starch films, it was reported that when the concentration of glycerol was increased (from 17 to 27%) the E% (from approximately 32 to 22%) and TS (from approximately 12 to 4 MPa) decreased [35], and although E% was higher than PoS (from 2.1 to 2.4%), the TS was lower than that obtained in this study for PoS films.

According to Figure 3, the tensile strength of PoS ranged from 21.2 to 30.4 MPa, similar to potato starch films with antioxidant and antimicrobial incorporation stored at 33% RH for 1 week. Protein-incorporated films showed TS values from 5 to 40 MPa, depending on the storage time and RH [32].

CaS C4-15 and C4-20 (32.6 and 32.2 MPa, respectively) showed the highest tensile strength, which was higher than that reported for cassava starch films at different sorbitol concentrations (about 2–30 MPa) [36], and it was reported that increasing the plasticizer concentration decreases the TS and increases the E%, because of the flexibility properties of the plasticizer [37,38]. For cassava starch films with gelatine and sorbitol, tensile strengths ranging from 70 to 170.31 MPa [12] were reported, which is higher than the results of CaS. According to statistical analyses for CaS, C3-17.5 (3 g·100 g^−1^ of water and 17.5% sorbitol) was different in TS and showed the lowest values for YM and E% and can be considered the worst formulation because of its rigid and weak behavior to external forces.

For the Young’s modulus, P4-20 was statistically different compared to the other films, being the lower value (1175.8 MPa). However, in general, all values showed characteristic of solid materials with low elasticity. A previous work reported that better mechanical and thermal properties could be achieved by the addition of citric acid and chitosan, with Young’s modulus ranging from 610 to 1024 MPa [21].

Comparing C4-15 and P4-15, C4-15 showed higher tensile strength (32.6 MPa). CaS films showed lower values of water content, solubility in water, and WVP than PoS films. Since water is a parameter that normally reduces the film’s TS by weakening the intermolecular forces [16], this explains why C4-15 was the film with higher TS.

For food packaging, low-density polyethylene (LDPE) is one of the most used materials. LDPE tensile strength ranges from 10 to 30 MPa depending on its thickness values [39]. Therefore, CaS and PoS films showed good resistance properties for food packaging when compared to LDPE tensile strength.

### 3.3. SEM, XRD and DSC Results

According to physicochemical and mechanical properties previously evaluated, C4-15 and P4-15 were the most suitable films materials for food packaging, and their structural and thermal properties were analyzed.

**Figure 4:**
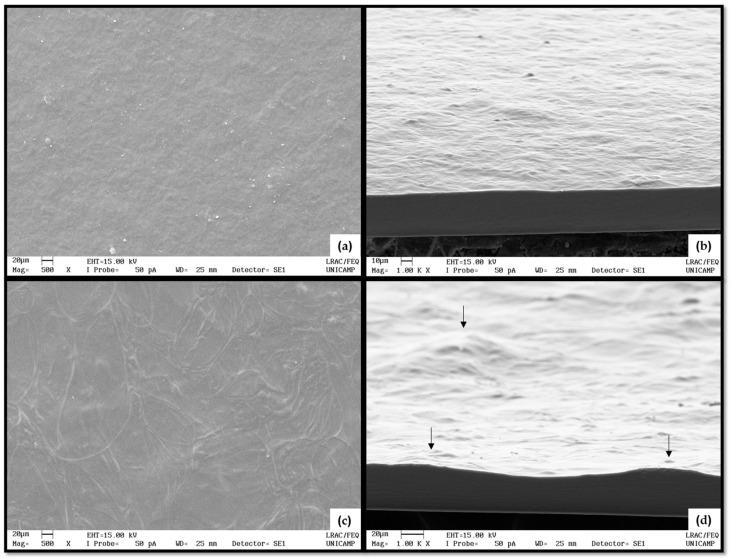
SEM of surfaces at (**a**,**c**) 500X and (**b**,**d**) cross-sections at 1000X of (**a**,**b**) C4-15 and (**c**,**d**) P4-15. Black arrows indicate some irregularities in relief (inequalities and elevations of the film surface or thickness).

The C4-15 and P4-15 surfaces and cross-sections were analyzed as shown in Figure 4. By the SEM images, it was possible to observe the structure and the structuring of the filmogenic matrix in each film, in which there were visible differences. In general, CaS and PoS showed a homogeneous surface, without bubbles or cracks. In Figure 4a,b, surfaces and cross-sections of C4-15 are shown. This film surface was similar to the one described by [40] for a modified cassava starch film of low viscosity.

However, P4-15 presented small irregularities elevations and inequalities in film surface and thickness as shown in Figure 4c,d, resulting in a non-uniform shape and size of the films as previously reported for native potato starch films [41]. The irregularities observed were attributed to the manufacturing process and could be adjusted using a vibrating table to better distribute the filmogenic solution homogeneously.

**Figure 5:**
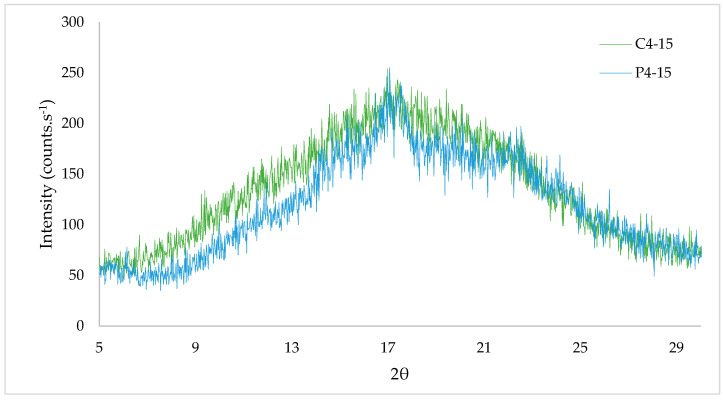
X-ray diffraction (XRD) of cassava (C4-15) and potato (P4-15) starch edible films.

**Figure 6:**
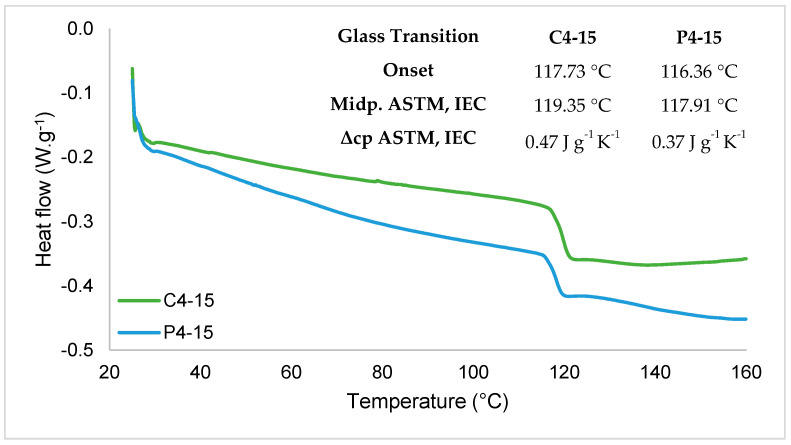
Differential scanning calorimetry thermogram (DSC) of cassava (C4-15) and potato (P4-15) starch edible films. Tg is the glass transition temperature, Midp. is the midpoint, and Δcp is the delta of specific heat capacity.

Figure 5 and Figure 6 show the XRD and DSC results, which also confirmed the homogeneity of the films. PoS films, specifically P4-15, were opaquer than CaS films. C4-15 resulted in a lighter material and more transparency than that previously reported for cassava starch films [42], and both PoS and CaS showed a white hue angle. Clear films are more accepted by consumers, although edible films can be clear or milky, but generally invisible [43]. Then, by visual parameters, C4-15 better suit a packaging material.

The cassava starch filmogenic solution was visibly less viscous than the potato starch filmogenic solution and was easier to homogenize. The potato starch had a most obvious change upon gelatinization, and the viscosity was increasing related to heating. Gelatinization steps can also be used to characterize starches [16].

The XRD allowed the observation of the CaS and PoS edible films’ crystalline regions. The peaks observed from 5 to 25° highlighted the crystalline region, and the peaks observed after 5°, 14°, 17° and between 21° and 25° matched with starches of B type, since at 5.6°, 14.4°, 17.2°, 22.2°, and 24.0°, strong bands appeared [44]. So, cassava and potato starches, as well as the films produced with them, had semi-crystalline morphology and behavior. However, the percentage of sorbitol added did not change crystallinity of cassava and potato starches. The behavior of crystalline regions’ formation and their intensity for both films were similar. Higher peaks were shown for cassava starch film (C4-15), but potato starch film (P4-15) showed the highest value at a point 17.07 of 2θ with an intensity of 255 counts·s^−1^ (Figure 5)

Previous studies have reported that characteristic diffraction peaks appeared clearly around 20° for potato starch films, noticing that glycerol increasing resulted in a crystallinity decrease [35,41].

The tendency to crystalline peaks around 20° for cassava starch films was also reported for cassava starch films with 30 and 45% of glycerol, coinciding with the C4-15 results [37].

The differential scanning calorimetry thermogram (Figure 6) showed that for the cassava and potato starch films, the glass transition occurred between 116.36 °C and 119.35 °C with higher values for the C4-15.

Still, that small difference could be because potato starch films had higher water content (15.95 g·100 g^−1^) than cassava starch films (12.38 g·100 g^−1^). The specific heat capacity value could explain why the filmogenic solution of potato starch showed a denser solution faster than the cassava starch filmogenic solution when subjected to the same temperature, as C4-15 had a higher specific heat capacity. The Tg values of C4-15 were similar to those reported by [37], who investigated the effect of glycerol content on the properties of cassava starch films that showed Tg values of 97.9 °C and 131.9 °C. They also observed a negative relation between Tg and glycerol content in which increasing glycerol content resulted in lower Tg values.

However, in the potato starch films case, higher values were previously reported with Tg ranging from 125.9 °C to 161.8 °C. Those values were related to different concentrations of antioxidant and antimicrobial proteins, although the film without protein incorporation showed a Tg of 125.9 °C, still higher than 117.91 °C of P4-15 Tg [32]. However, the cassava and potato starch films showed good workability, an organized structure, and strong mechanical properties, but thermal degradation could be improved in order to allow the thermoprocessing of the materials for food packaging development.

Therefore, cassava and potato starch offer significant advantages for food products due to their physicochemical, thermal, and mechanical properties, which facilitate their integration with other compounds [12,32,34]. And as plasticizer to reinforce the film formation, sorbitol is an edible carbohydrate widely used in the food industry and can provide sweetness, texture, and moisture retention [45].

In addition to all the properties analyzed above, it is important to highlight that films produced from potato and cassava starch can be degraded under different composting conditions [4], meaning an important contribution to the reduction of contamination caused by plastic packaging and reinforcing the green economy.

## 4. Conclusions

Cassava and potato starch are optimal alternatives for integrating the formulation of edible films for food packaging because of their compatibility with edible ingredients and their high degradation rate. As discussed, those starches could interact with multiple compounds in order to add functional properties to the packaging or to improve the mechanical characteristics.

According to the CCRD predictive mathematical models for cassava starch films, it was observed that the addition of sorbitol significantly influenced the WVP and the solubility for the potato starch films. The addition of sorbitol showed an influence in the water content, WVP, thickness, and a_w_. For CaS films, it was observed that low WVP (around 4 to 6 g·mm/m^2^) and solubility between 10 and 15 g·100 g^−1^ were obtained in the starch concentration of 4 g·100 mL^−1^ of water with 15–20% sorbitol, considering those values to optimal formulations. PoS CCRD made it possible to consider more parameters for optimal formulation; then, WVP 6 g·mm/m^2^, 0.04 mm of thickness, water content around 15–20 g·100 g^−1^, and an a_w_ under 0.5 were also obtained in the region of 4 g·100 mL^−1^ of water with 15–20% sorbitol.

Both cassava and potato starch films were resistant and homogeneous with similar mechanical, structural, and thermal properties behavior. The tensile strength of both materials was high, coinciding with rigid films. As they are starches, the CaS and PoS films showed a semi-crystalline morphology and Tg above 100 °C. The scanning electron micrographs revealed that both films had a homogeneous surface without bubbles or cracks. In summary, cassava and potato starch films have suitable properties for edible films for food packaging and in this way contribute to the reduction of plastic use in the food industry.

## Figures and Tables

**Table 1:** Encoded and real values for CCRD.

Experimental Run	Encoded Starch Concentration (x)	Encoded Plasticizer Percentage (y)	Real x (g·100 mL^−1^ of Water)	Real y (%)
1	−1.00	−1.00	2.00	15.0
2	−1.00	1.00	2.00	20.0
3	1.00	−1.00	4.00	15.0
4	1.00	1.00	4.00	20.0
5	−1.41	0.00	1.59	17.5
6	1.41	0.00	4.41	17.5
7	0.00	−1.41	3.00	14.0
8	0.00	1.41	3.00	21.0
9	0.00	0.00	3.00	17.5
10	0.00	0.00	3.00	17.5
11	0.00	0.00	3.00	17.5

**Table 2:** CCRD experimental results and physicochemical characterization of PoS and CaS films. The values are the means of the experimental data and the standard deviation.

**PoS**					
**Experimental Run**	**Water Content (g·100 g^−1^)**	**Solubility (g·100 g^−1^)**	**a_w_** **(Decimal)**	**WVP (g·mm/m^2^ day·kPa)**	**Thickness (mm)**
1	20.36 ± 7.75	17.74 ± 3.67	0.539 ± 0.013	4.39 ± 0.07	0.033 ± 0.004
2	27.06 ± 1.51	20.17 ± 1.77	0.493 ± 0.008	3.12 ± 0.17	0.032 ± 0.006
3	15.95 ± 0.92	11.48 ± 3.66	0.436 ± 0.012	4.86 ± 0.08	0.068 ± 0.017
4	10.32 ± 3.87	5.29 ± 1.06	0.429 ± 0.008	5.82 ± 0.53	0.049 ± 0.003
5	29.94 ± 3.09	16.90 ± 0.91	0.538 ± 0.006	4.33 ± 0.47	0.024 ± 0.004
6	12.11 ± 1.46	3.13 ± 0.44	0.416 ± 0.005	2.64 ± 0.26	0.057 ± 0.004
7	23.35 ± 1.38	11.90 ± 0.23	0.517 ± 0.005	6.80 ± 0.22	0.042 ± 0.002
8	19.63 ± 0.75	12.33 ± 0.16	0.518 ± 0.005	4.70 ± 0.15	0.042 ± 0.002
9	18.26 ± 3.74	15.76 ± 0.58	0.517 ± 0.007	5.78 ± 0.03	0.045 ± 0.004
10	21.62 ± 4.63	16.27 ± 2.19	0.497 ± 0.002	5.68 ± 0.56	0.045 ± 0.004
11	20.33 ± 2.15	24.04 ± 0.31	0.516 ± 0.009	6.31 ± 0.19	0.042 ± 0.002
**CaS**					
**Experimental Run**	**Water Content (g·100 g^−1^)**	**Solubility (g·100 g^−1^)**	**a_w_** **(Decimal)**	**WVP (g·mm/m^2^ day·kPa)**	**Thickness (mm)**
1	13.65 ± 1.44	9.96 ± 1.57	0.440 ± 0.006	8.02 ± 0.89	0.034 ± 0.005
2	13.26 ± 3.27	16.53 ± 1.84	0.441 ± 0.007	2.95 ± 0.27	0.030 ± 0.002
3	12.38 ± 2.11	5.41 ± 0.70	0.533 ± 0.004	3.26 ± 0.52	0.030 ± 0.005
4	15.47 ± 1.13	11.35 ± 1.64	0.529 ± 0.011	2.27 ± 0.06	0.032 ± 0.003
5	7.95 ± 1.78	10.50 ± 0.46	0.489 ± 0.011	4.42 ± 0.15	0.023 ± 0.002
6	15.03 ± 0.95	11.04 ± 0.29	0.436 ± 0.004	3.33 ± 0.66	0.057 ± 0.006
7	4.10 ± 0.73	15.73 ± 0.96	0.471 ± 0.004	3.80 ± 0.31	0.036 ± 0.008
8	6.19 ± 0.22	18.39 ± 1.77	0.464 ± 0.022	3.24 ± 0.18	0.032 ± 0.004
9	10.21 ± 1.52	5.44 ± 2.77	0.457 ± 0.011	2.42 ± 0.18	0.024 ± 0.000
10	11.45 ± 0.61	3.39 ± 0.25	0.429 ± 0.008	2.42 ± 0.19	0.032 ± 0.002
11	11.40 ± 0.72	3.06 ± 0.46	0.447 ± 0.005	2.40 ± 0.59	0.034 ± 0.003

**Table 3:** ANOVA table for potato starch films results—WVP, thickness, water content, and a_w_.

	SS	df	MS	Fcalc	Ftab _(10%)_
WVP					
Regression	11.58	3	3.86	5.27	3.07
Residue	5.13	7	0.73		
Lack of Fit	4.89	5	0.98	8.52	9.29
Pure error	0.23	2	0.12		
Total	16.71	10			
Thickness					
Regression	0.0013	3	0.00045	33.37	3.07
Residue	9.39 × 10^−5^	7	1.34 × 10^−5^		
Lack of Fit	0.00009	5	1.75 × 10^−5^	5.41	9.29
Pure error	0.00001	2	3.23 × 10^−6^		
Total	0.00144	10			
Water content					
Regression	306.58	2	153.29	39.03	3.11
Residue	31.42	8	3.93		
Lack of Fit	25.67	6	4.23	1.49	9.33
Pure error	5.74	2	2.87		
Total	337.995	10			
a_w_					
Regression	0.0173	2	0.0086564	28.14	3.11
Residue	0.0025	8	0.0003076		
Lack of Fit	0.0022	6	0.000367	2.84	9.33
Pure error	0.0003	2	0.000129		
Total	0.0198	10			

**Table 4:** ANOVA table for cassava starch films results—WVP and solubility.

	SS	df	MS	Fcalc	Ftab _(10%)_
WVP					
Regression	21.15	5	4.23	3.69	3.45
Residue	5.74	5	1.15		
Lack of Fit	5.74	3	1.91	9180.07	9.16
Pure error	0.00042	2	0.00021		
Total	26.89	10			
Solubility					
Regression	226.55	3	75.52	9.44	3.07
Residue	56.01	7	8.002		
Lack of Fit	52.69	5	10.54	6.34	9.29
Pure error	3.33	2	1.66		
Total	282.56	10			

## Data Availability

The data presented in this study are available on request from the corresponding author.

## References

[1] Brandão A.S., Gonçalves A., Santos J.M.R.C.A. (2021). Circular bioeconomy strategies: From scientific research to commercially viable products. J. Clean Prod..

[2] Punnagaiarasi A., Elango A., Rajarajan G., Prakash S., Prashanthi M., Sundaram R., Jeyaseelan A., Kaliannan T. (2017). Application of Bioremediation on Food Waste Management for Cleaner Environment. Bioremediation and Sustainable Technologies for Cleaner Environment.

[3] Walker T.R., Fequet L. (2023). Current trends of unsustainable plastic production and micro(nano)plastic pollution. TrAC Trends Anal. Chem..

[4] Rai P., Mehrotra S., Priya S., Gnansounou E., Sharma S.K. (2021). Recent advances in the sustainable design and applications of biodegradable polymers. Bioresour. Technol..

[5] Kumar G.M., Irshad A.V.B.R., Rajarajan G. (2016). Waste Management in Food Packaging Industry. Integrated Waste Management in India Prashanthi, M., Sundaram, R., Eds..

[6] Adilah A.N., Jamilah B., Noranizan M.A., Hanani Z.A.N. (2018). Utilization of mango peel extracts on the biodegradable films for active packaging. Food Packag. Shelf. Life.

[7] Hanani Z.A.N., Husna A.B.A., Syahida S.N., Khaizura M.A.B.N., Jamilah B. (2018). Effect of different fruit peels on the functional properties of gelatin/polyethylene bilayer films for active packaging. Food Packag. Shelf. Life.

[8] Ellen MacArthur Foundation (2019). Cities and Circular Economy for Food. https://www.ellenmacarthurfoundation.org/cities-and-circular-economy-for-food.

[9] Sharma S., Basu S., Shetti N.P., Aminabhavi T.M. (2020). Waste-to-energy nexus for circular economy and environmental protection: Recent trends in hydrogen energy. Sci. Total Environ..

[10] Baldissera A.C., Betta F.D., Penna A.L., Lindner J.D. (2011). Alimentos funcionais: Uma nova fronteira para o desenvolvimento de bebidas protéicas a base de soro de leite. Semina Ciênc. Agr..

[11] Barandiaran A., Montanes N., Gomez-Caturla J., Balart R., Florez-Prieto M.A., Ávila-Martin L., Perilla J.E. (2024). Development and characterization of edible films based on starch isolated from different Colombian potato varieties. Int. J. Biol. Macromol..

[12] Fakhouri F.M., Martelli S.M., Bertan L.C., Yamashita F., Mei L.H., Queiroz F.P. (2012). Edible films made from blends of manioc starch and gelatine Influence of different types of plasticizer and different levels of macromolecules on their properties. LWT-Food Sci. Technol..

[13] Rojas-Bringas P.M., De-La-Torre G.E., and Torres F.G. (2021). Influence of the source of starch and plasticizers on the environmental burden of starch-Brazil nut fiber biocomposite production: A life cycle assessment approach. Sci. Total Environ..

[14] Udayakumar G.P., Mathusamy S., Selvaganesh B., Sivarajasekar N., Rambabu K., Banat F., Sivamani S., Sivakumar N., Hosseini-Bandegharaei A., Show P.L. (2021). Biopolymers and composites: Properties, characterization and their applications in food, medical and pharmaceutical industries. J. Environ. Chem. Eng..

[15] Huber K.C., Embuscado M.E. (2009). Edible Films and Coatings for Food Applications.

[16] Kramer M.E., Huber K., Embuscado M. (2009). Structure and Function of Starch-Based Edible Films and Coatings. Edible Films and Coatings for Food Applications.

[17] Fakhouri F.M., Martelli S.M., Caon T., Velasco J.I., Mei L.H. (2015). Edible films and coatings based on starch/gelatin: Films properties and effect of coatings on quality of refrigerated Red Crissom grapes. Postharvest Biol. Technol..

[18] Han B., Chen P., Guo J., Yu H., Zhong S., Li D., Liu C., Feng Z., Jiang B. (2023). A Novel Intelligent Indicator Film: Preparation, Characterization, and Application. Molecules.

[19] Filgueiras C.T., Fakhouri F.M., Garcia V.A.d.S., Velasco J.I., Nogueira G.F., Ramos da Silva L., Oliveira R.A.d. (2024). Effect of Adding Red Propolis to Edible Biodegradable Protein Films for Coating Grapes: Shelf Life and Sensory Analysis. Polymers.

[20] Rodrigues G.M., Filgueiras C.T., Gracia V.A.S., Carvalho R.A., Velasco J.I., Fakhouri F.M. (2020). Antimicrobial Activity and GC-MS Profile of Copaiba Oil for Incorporation into Xanthosoma mafaffa Schott Starch-Based Films. Polymers.

[21] Hernández M.S., Ludueña L.N., Flore S.K. (2023). Citric acid, chitosan and oregano essential oil impact on physical and antimicrobial properties of cassava starch films. Carbohydr. Polym. Technol. Appl..

[22] Matheus J.R.V., Farias P.M., Satoriva J.M., Andrade C.J., Fai A.E.C. (2023). Cassava starch films for food packaging: Trends over the last decade and future research. Int. J. Biol. Macromol..

[23] Bertoft E., Blennow A., Singh J., Kaur L. (2016). Chapter 3–Structure of potato starch. Advances in Potato Chemistry and Technology.

[24] Helrich K., AOAC-Association of Analytical Chemists (1990). Official Methods of Analysis of the Association of Analytical Chemists.

[25] Gontard N., Guilbert S., Cuq J.L. (1992). Edible Wheat Gluten Films: Influence of the Main Process Variables on Film Properties using Response Surface Methodology. J. Food Sci..

[26] ASTM-American Society for Testing and Materials, Subcommittee C16.33 (2017). Standard Test Methods for Water Vapor Transmission of Materials (ASTM –E9600e1). Book of Standards.

[27] Mchugh T.H., and Krochta J.M., Krochta J.M., Baldwin E.A., Nisperos-Carriedo M. (1994). Permeability properties of edible films. Edible Coatings and Films to Improve Food Quality.

[28] ASTM-American Society for Testing and Materials, Subcommittee D20.19 (2018). Standard test method for tensile properties of thin plastic sheeting (ASTM -D882-18). Book of Standards.

[29] Sartori T., Menegalli F.C. (2016). Development and characterization of unripe banana starch films incorporated with solid lipid microparticles containing ascorbic acid. Food Hydrocoll..

[30] Fakhouri F.M., Fontes L.C., Gonçalves P.V., Milanez C.R., Steel C.J., Collares-Queiroz F.P. (2007). Films and edible coatings based on native starches and gelatin in the conservation and sensory acceptance of Crimson grapes. Food Sci. Technol..

[31] Zavareze E.R., Onto V.Z., Klein B., Halal S.L., Elias M.C., Prentice-Hernández C., Dias A.R. (2012). Development of oxidised and heat–moisture treated potato starch film. Food Chem..

[32] Moreno O., Atarés L., Chiralt A. (2015). Effect of the incorporation of antimicrobial/antioxidant proteins on the properties of potato starch films. Carbohydr. Polym..

[33] Caetano K.S., Lopes N.A., Costa T.M.H., Brandelli A., Rodrigues E., Flôres S.H., Cladera-Olivera F. (2018). Characterization of active biodegradable films based on cassava starch and natural compunds. Food Packag. Shelf. Life..

[34] Farias M.G., Fakhouri F.M., Piler C.W., Ascheri J.L. (2012). Physicochemical characterization of edible starch films with barbados cherry (Malphigia emarginata D.C.). Quím. Nova..

[35] Hu G., Chen J., Gao J. (2009). Preparation and characteristics of oxidized potato starch films. Carbohydr. Polym..

[36] Shimazu A.A., Mali S., Grossmann M.V. (2007). Plasticizing and antiplasticizing effects of glycerol and sorbitol on biodegradable cassava starch films. Semina Ciênc. Agr..

[37] Bergo P.V., Carvalho R.A., Sobral P.J., Dos Santos R.M., Da Silva F.B., Prison J.M., Solorza-Feira J., Habitante A.M. (2008). Physical Properties of Edible Films Based on Cassava Starch as Affected by the Plasticizer Concentration. Packag. Technol. Sci..

[38] Lagos J.B., Vicentini N.M., Dos Santos R.M., Bittante A.M., Sobral P.J. (2015). Mechanical properties of cassava starch affected by different plasticizers and different relative humidity conditions. Int. J. Food Stud..

[39] Rennert M., Nase M., Lach R., Reinck K., Arndt S., Androsch R., Grellmann W. (2013). Influence of low-density polyethylene blown film thickness on the mechanical properties and fracture toughness. J. Plas. Film Sheeting.

[40] Henrique C.M., Cereda M.P., Sarmento S.B. (2008). Características físicas de filmes biodegradáveis produzidos a partir de amidos modificados de mandioca. Food Sci. Technol..

[41] Zhang R., Wang X., Cheng M. (2018). Preparation and Characterization of Potato Starch Film with Various Size of Nano-SiO_2_. Polymers.

[42] Lim W.S., Ock S.Y., Park G.D., Lee I.W., Lee M.H., Park H.J. (2020). Heat-sealing property of cassava starch film plasticized with glycerol and sorbitol. Food Packag. Shelf Life.

[43] Pavlath A.E., Orts W., Huber K., Embuscado M. (2009). Edible Films and Coatings: Why, What, and How. Edible Films and Coatings for Food Applications.

[44] Zobel H.F., Alexander R.J. (1994). Starch granule structure. Developments in Carbohydrate Chemistry.

[45] Sravan T., Spandana K. (2021). Sorbitol–Its applications in different fields. Agric. Food E-Newsl..

